# Prolonged grief during and beyond the pandemic: factors associated with levels of grief in a four time-point longitudinal survey of people bereaved in the first year of the COVID-19 pandemic

**DOI:** 10.3389/fpubh.2023.1215881

**Published:** 2023-09-19

**Authors:** Emily Harrop, Renata Medeiros Mirra, Silvia Goss, Mirella Longo, Anthony Byrne, Damian J. J. Farnell, Kathy Seddon, Alison Penny, Linda Machin, Stephanie Sivell, Lucy E. Selman

**Affiliations:** ^1^Division of Population Medicine, Marie Curie Research Centre, Cardiff University, Cardiff, United Kingdom; ^2^School of Dentistry, Cardiff University, Cardiff, United Kingdom; ^3^National Bereavement Alliance, London, United Kingdom; ^4^School of Medicine, Keele University, Keele, United Kingdom; ^5^Palliative and End of Life Care Research Group, Population Health Sciences, Bristol Medical School, University of Bristol, Bristol, United Kingdom

**Keywords:** bereavement, longitudinal, COVID-19, COVID-19 pandemic, grief, UK

## Abstract

**Background:**

The COVID-19 pandemic has been a devastating and enduring mass-bereavement event, with uniquely difficult sets of circumstances experienced by people bereaved at this time. However, little is known about the long-term consequences of these experiences, including the prevalence of Prolonged Grief Disorder (PGD) and other conditions in pandemic-bereaved populations.

**Methods:**

A longitudinal survey of people bereaved in the UK between 16 March 2020 and 2 January 2021, with data collected at baseline (*n* = 711), c. 8 (*n* = 383), 13 (*n* = 295), and 25 (*n* = 185) months post-bereavement. Using measures of Prolonged Grief Disorder (PGD) (Traumatic Grief Inventory), grief vulnerability (Adult Attitude to Grief Scale), and social support (Inventory of Social Support), this analysis examines how participant characteristics, characteristics of the deceased and pandemic-related circumstances (e.g., restricted visiting, social isolation, social support) are associated with grief outcomes, with a focus on symptoms of PGD.

**Results:**

At baseline, 628 (88.6%) of participants were female, with a mean age of 49.5 (SD 12.9). 311 (43.8%) deaths were from confirmed/suspected COVID-19. Sample demographics were relatively stable across time points. 34.6% of participants met the cut-off for indicated PGD at c. 13 months bereaved and 28.6% at final follow-up. Social isolation and loneliness in early bereavement and lack of social support over time strongly contributed to higher levels of prolonged grief symptoms, while feeling well supported by healthcare professionals following the death was associated with reduced levels of prolonged grief symptoms. Characteristics of the deceased most strongly associated with lower levels of prolonged grief symptoms, were a more distant relationship (e.g., death of a grandparent), an expected death and death occurring in a care-home. Participant characteristics associated with higher levels of prolonged grief symptoms included low level of formal education and existence of medical conditions.

**Conclusion:**

Results suggest higher than expected levels of PGD compared with pre-pandemic times, with important implications for bereavement policy, provision and practice now (e.g., strengthening of social and specialist support) and in preparedness for future pandemics and mass-bereavement events (e.g., guidance on infection control measures and rapid support responses).

## 1. Background

Millions of people were bereaved during the COVID-19 pandemic, with close to seven million reported deaths caused by the virus world-wide, and over 200,000 in the UK (1). This prolonged mass-bereavement event was characterized by high death-rates and unprecedented restrictions to usual end-of-life, death and mourning practices and social life in general. In the early months of the pandemic, observers predicted worsened grief and bereavement outcomes in response to the sudden and unexpected nature of COVID-19 deaths, the traumatic circumstances in which these deaths occurred and the likely diminished coping capacities of bereaved people (and the people and services supporting them) (2–4).

More than 3 years on from the start of the global pandemic, there is now a considerable body of evidence documenting the impacts of these devastating, unique sets of circumstances on those bereaved at this time. However, little is currently known about the longer-term consequences of pandemic bereavement, including which groups of people are most at risk of adverse outcomes over time and whether initial predictions of increased levels of Prolonged Grief Disorder (PGD) are substantiated (2–4). Although it is generally expected that most bereaved individuals will adequately cope with their grief and slowly readjust to life without the deceased, it is recognized that a significant minority of bereaved individuals will experience more complicated and problematic grieving processes, including development of PGD (5, 6). Essential characteristics of PGD include persistent and pervasive longing for, or preoccupation with, the deceased, associated with intense emotional pain (e.g., sadness, guilt, denial), functional impairment, and atypically prolonged symptoms relative to cultural norms (lasting a minimum of 6 months post-bereavement) (5–8). Although figures on PGD or complex grief vary between studies [e.g., between 6 and 20%; (5, 6, 9, 10)] in non-pandemic public health models, it is commonly accepted that around 10% of bereaved people will experience PGD, requiring specialist psychological intervention, while those with “moderate” level needs and risk (estimated at around 30%) may also need formal bereavement support such as peer-support groups or grief counseling (9, 11). Evidence on PGD levels during and following the pandemic is therefore needed to better understand the long-term grief and associated support-needs of people bereaved during this and future pandemics, with implications for bereavement service-planning and delivery. It is also critical for informing policy considerations relating to infection-control isolation measures in both the current COVID-19 recovery phase, and as part of our preparedness for future outbreaks of infectious diseases.

Most evidence to date on the grief and mental health consequences of pandemic bereavement is from studies conducted in China (12, 13), North America (14–18), Holland (4, 19, 20) and the earlier qualitative and quantitative results from this UK-based study (21–26). Several of these cross-sectional studies [including our baseline publication, (23)] indicate higher levels of grief and functional impairment among people bereaved during the pandemic, compared with pre-pandemic populations (14–20). Many of these studies have demonstrated the negative grief impacts of pandemic-specific or related circumstances. These have included restricted visiting at the end of life and opportunities to say goodbye (15, 17, 20, 24), sub-optimal communication and support from healthcare staff at the end of life (17, 22, 24), disrupted funerals (17, 23, 24), experiences of loneliness and isolation (8, 15, 17, 23, 24) and the role of disrupted meaning-making in mediating the effects of these sets of circumstances (15, 17). Another study, by contrast, found no differences in levels of PGD, attendance at, or evaluations of funerals and other mourning rituals, between pandemic and pre-pandemic bereaved populations (27).

Of particular interest early on in the pandemic was whether COVID-19 deaths would be associated with worse grief experiences than other types of death. Higher levels of grief and other psychological conditions have been identified among those bereaved by COVID-19 than would be expected in non-pandemic populations (12–14, 17), or compared with “natural” but not “unnatural” causes of deaths pre and during the pandemic (4, 18–20), with the “unexpected” nature of these deaths an explanatory factor (19, 20). However, other studies (including the baseline results from this study) have not found significant differences in grief and other psychological outcomes between COVID-19 and non-COVID-19 bereavement during the pandemic (15, 23). Several of these studies have also investigated the effects of demographic and other known risk factors for adverse grief and health outcomes. Consistent with pandemic (12–14, 28, 29) and non-pandemic research (9, 30), our baseline results identified relationship with the deceased as the strongest factor predicting grief vulnerability (23). Younger age of the deceased was also associated with worse baseline grief vulnerability (23), as in other studies (9, 29, 31). Age, gender, race/ethnicity of the bereaved person, and time since death were not significantly associated with level of grief in our baseline results (23) or functional impairment in one of the US studies (14). By contrast, lower levels of education were associated with poorer outcomes in our baseline results (23), reflecting the findings of previous non-pandemic research (32–34). Related associations with low income have also been identified, including a study involving pandemic and pre-pandemic bereaved participants (8).

However, to date no longitudinal results that we know of have been published on grief outcomes during the pandemic, and most of the above mentioned studies included pandemic-bereaved populations who were on average bereaved less than 6 months before [e.g., (4, 12, 14, 15, 17, 19, 20, 23)], thus limiting observations that can be made regarding levels of, and factors associated with, PGD symptoms in their respective populations. Addressing this knowledge gap, this paper reports longitudinal results regarding factors associated with PGD symptoms among a cohort of participants bereaved during the first two waves of the pandemic in the UK, using data collected at four time-point survey rounds, up to 25 months post-bereavement.

## 2. Methods

### 2.1. Study design and aim

A longitudinal survey of people bereaved during the pandemic in the UK. The web-based survey was conducted as part of a larger mixed methods study, which aimed to investigate the grief experiences, support needs and use of bereavement support by people bereaved during the pandemic (21–26). The current analysis examines how clinical and demographic factors, and pandemic-related challenges are associated with symptoms of PGD in a cohort of participants surveyed at baseline (T1) and c. 8, 13, and 25 months post-bereavement (T2–T4). The mediating role of perceived social support was also investigated in this analysis, reflecting its established importance for healthy grieving and adaptation [e.g., (10, 35–37)] and its likely association with other demographic factors potentially also predictive of grief severity (e.g., gender, ethnicity, age).

The Checklist for Reporting Results of Internet E-Surveys (38) was followed.

### 2.2. Survey development

An open web survey was designed by the research team, which includes a public representative (KS), with input from the study advisory group. Each survey was piloted, refined with public representatives with experience of bereavement, and tested by the study advisory group and colleagues. Non-randomized open and closed questions covered end of life experiences, grief experiences, and perceived needs for, access to and experiences of formal and informal bereavement support (21, 22).

### 2.3. Outcome measures

*Prolonged Grief Disorder* was assessed at surveys T2-T4 using the Traumatic Grief Inventory Self-Report version (TGI-SR) (39, 40). This widely used 18-item self-report measure assesses symptoms of Persistent Complex Bereavement Disorder (PCBD) and PGD, as defined by Prigerson et al. (6). The TGI-SR includes all 16 symptoms of PCBD, one additional symptom of PGD that is not part of the PCBD criteria (i.e., item 12: “feeling stunned/shocked”) and one item tapping “functional impairment” (i.e., item 13), included in criteria-sets for both PCBD and PGD (40). Participants rated the frequency of symptoms (e.g., “I felt a strong longing or yearning for the deceased”) during the previous month on 5-point scales (1 = never and 5 = always). Total scores ranged from 16 to 80. A cut-off score of ≥54 (i.e., mean item score of 3.0) is indicative of PCBD and PGD when using the total score (40). The measure was not used at baseline as PGD should be assessed at least 6 months after a death, and PCBD at least 12 months afterwards (39, 40).

*Vulnerability in Grief* was assessed in all survey time points using the validated 9-item Adult Attitude to Grief (AAG) scale (41), with our reasons for selecting this measure reported in baseline publications (21, 23). The scale is based on the Range of Response to Loss model (42), which identifies three distinct responses: being “overwhelmed,” a state dominated by emotional/cognitive distress; being “controlled,” needing to avoid emotional expression and focus on day-to-day life; and being balanced or “resilient,” feeling supported and able to cope. AAG subscale scores indicate levels of feeling overwhelmed, controlled, and reversed resilience on a scale of 0 (none) to 12 (very high). An overall index of vulnerability (IoV) is calculated by summing subscale scores [IoV: 0–20 = low vulnerability, 21–23 = high vulnerability, and 24–36 = severe vulnerability (41)]. Although this analysis and publication is focused on symptoms of PGD, we included this measure to enable comparisons to be made with our analysis of baseline survey data, which did not include the TGI measure (23).

*Social support* was assessed using the Inventory of Social Support (ISS) (43). The ISS is a 5-item measure that assesses how far a bereaved person can talk with other people about their loss in a way which supports adaptive coping. The measure includes such statements as “I can express my feelings about my grief openly and honestly” “There is at least one person I can talk to about my grief.” Participants respond to such statements on a 5-point Likert scale that ranges from 1 (Does not describe me very well) to 5 (Describes me very well). Higher scores on the ISS indicate higher levels of social support. Social support was investigated both as a dependent variable and as an independent variable in the PGD/TGI model.

### 2.4. Associated factors

We assessed whether participant characteristics and characteristics of the deceased, experiences of end-of-life care and pandemic-related problems independently predicted levels of PGD symptoms and grief vulnerability and whether perceived social support mediated the relationships of these variables and symptoms of PGD. Factors included in the analysis are recognized risk factors for poor bereavement outcomes (age of deceased and bereaved, gender, time since death, relationship to deceased, expectedness of the death, ability to say goodbye to the deceased, support from healthcare professionals at the end of life, perceived social support) (10, 23, 44–46) or are known to be indirectly associated with such outcomes (qualifications, health status, place of death, cause of death) (47, 48).

Six items at baseline assessed pandemic-related challenges prior to and after the death, e.g., being unable to visit the person who died prior to their death, restricted funeral arrangements, social isolation and loneliness. All items were answered yes/no. Respondents were asked to tick all experiences that applied to them.

See Supplementary Files 1, 2 for baseline and final questionnaires, including all measures used in this analysis.

### 2.5. Study procedure

The baseline survey was administered via JISC^1^ and was open from 28th August 2020 to 5th January 2021 (21–24). It was disseminated to a convenience sample from social and mainstream media and via voluntary sector associations and bereavement support organizations, including those working with ethnic minority communities. Organizations helped disseminate the voluntary (non-incentivized) survey by sharing on social media, web-pages, newsletters, on-line forums and via direct invitations to potential participants. For ease of access, the survey was posted onto a bespoke study-specific website with a memorable URL.^2^ Hard-copy postal surveys were available on request. The second, third and fourth follow up surveys were sent to baseline participants who consented to receive follow up surveys around seven, 13 and 25 months post date of death. These were personalized for each participant using individual survey links, labeled with their participant study IDs. Where baseline surveys were completed at least 5 months post-death (or the date of death was not given), the second survey was sent out 2 months after the first survey was received. All second-round surveys were completed between 20/11/20 and 24/08/2021 and on average 242 days (median = 234 days or 8 months) after the date of death (range 145 to 345 days). All third-round surveys were completed between 04/05/2021 and 09/01/22 and on average 408 days (median = 404 or 13 months) after the date of death (range 396–481 days). All fourth round surveys were completed between 17/05/2022 and 12/01/2023, on average 776 days (median = 774 or 25 months) after the date of death (range 762–812 days).

Inclusion criteria for study enrolment: aged 18+; family member or close friend bereaved since social-distancing requirements were introduced in the UK (16/03/2020); death occurred in the UK; ability to consent. The initial section of the survey requested informed consent and details data protection.

### 2.6. Data analysis

All analysis was performed using R (version 4.1.1), implemented in R-Studio^3^ (49). Descriptive statistics were used to describe all variables. The main outcome variable of interest in this study was levels of PGD symptoms, assessed through the TGI questionnaire. ISS scores were used both as a predictor of levels of PGD symptoms and as an outcome variable, indirectly exploring the potential mediation effect of ISS between some of the independent variables and PGD levels. Since TGI and ISS scores were not collected at baseline, IoV scores for the AAG questionnaire were also used as an outcome variable to allow for comparisons between baseline and the other time points. Mean imputation was used to replace missing values in the AAG questionnaire for each sub-category if two of the three scores were available; IoV scores were used only if data were available for all three sub-categories. Mean imputation was also used to replace missing values for ISS and TGI scores. No more than 2% of data points were mean imputed for AAG and ISS at each round of surveys (including zero imputations for AAG at T4) and less than 5% of data points were mean imputed for TGI at each round of surveys. Most participants only had one missing value imputed and the maximum number of imputations for the same participant was three for TGI and ISS and two for AAG. The thresholds used for IoV categories followed Sim et al. (41) and the threshold used for PCBD and PGD was TGI score ≥ 54 (40).

Independent variables were classified into three categories: characteristics of the participant/bereaved, characteristics of the deceased, and characteristics of the experience of bereavement. The latter included the six items assessing the pandemic-related challenges prior to and after the death as well as whether participants felt well supported by healthcare professionals immediately after the death. For the analysis of levels of PGD symptoms, ISS was also included in this category.

Independent variables with more than 5% missing data at any of the four time points were not considered for analysis. A summary analysis of missing data was carried out for the remaining variables to check if there were any obvious patterns of missingness and none were identified. In order to maximize the sample, results presented are from analysis carried on all data available for each variable or combination of variables used in each analysis, but all the analyses were also carried out using complete cases (i.e., excluding any participants with at least one missing data for any of the variables of interest) as a control to ensure that the missing data were not causing a great influence in the results.

Days since bereavement was scaled into z-scores due to the wide range of values. This variable was initially tested both as a linear and as a quadratic term, but the latter showed very small effects and did not improve the fit of the statistical models significantly and hence the scaled linear effect was used instead. Other genders besides male or female were not included in the analysis due to the very small sample sizes. For relationship with the deceased, the categories “other family member” and “colleague or friend” were merged into one category in the analysis. Likewise, for place of death, the categories “other” and “do not know” were also merged. The existence of any medical conditions, whether the bereaved respondent was unemployed during the pandemic and whether they had suffered any further bereavements throughout the study were considered cumulatively, i.e., if a participant had reported a medical condition, becoming unemployed or suffering a further bereavement in one round of the survey, that was carried through even though they might not have reported it again in a subsequent round.

The first step of the analysis consisted of fitting Linear Mixed Models (LMMs) to assess the single effects of each independent variable on each of the outcome variables (IoV, ISS, and TGI scores). The second step consisted of fitting LMMs for each group of variables in combination to assess which group of variables (characteristics of the participant/bereaved, characteristics of the deceased, or characteristics of the experience of bereavement) were better at explaining each of the outcome variables.

The third step consisted of fitting LMMs that included all groups of variables in combination to assess the effect of the experience of bereavement in each of the outcome variables, while controlling for the characteristics of the participant/bereaved and the characteristics of the deceased. These models failed to converge and, hence, a Principal Component Analysis (PCA) was carried out on the six items assessing the pandemic-related challenges prior to and after the death, to assess if these could be reduced to a smaller number of factors. The models were initially run with the factors from the PCA, instead of the six different items. Variables with negligible effect sizes were then removed and the factors were replaced by the six items in the reduced model. The final models were used to compute and plot predictions showing the change of IoV, ISS, and TGI scores across time for the different levels of the independent variables that showed medium or strong effect sizes. For the TGI (symptoms of PGD), two different models were fit, one with ISS as predictor and one without, to assess the role of ISS as a potential mediator of other predictors.

All models included participant ID as random term and days since bereavement as a covariate. Interactions between days since bereavement and all other variables were tested at this stage. Statistically significant interactions (*p* < 0.05) found in the single models were also tested in the final models. Model estimates and standardized effect sizes were used to evaluate the effect of each variable independently on IoV, ISS, and TGI scores; Cohen’s *d* were used for categorical predictors: *d* = 0.3: small effect, *d* = 0.5: medium effect, *d* = 0.8: large effect, *d* = 1.2: very large; and partial R for continuous predictors: <0.10: trivial effect, 0.1–0.3: small to medium effect, 0.3–0.5: medium to large effect, >0.50: large to very large effect (50). Where categorical predictors contained more than one group, we chose a reference category that allowed us to show the maximum difference in means between any two groups and the average standard deviation across all groups (i.e., maximum effect size of the difference). By using a standardized measure of effect size, the effects of factors on outcomes could be compared directly and patterns across multiple outcomes ascertained. Marginal R^2^ (51) were used to assess the overall fit of the models in terms of their explanatory power and to explore which variables were the greatest contributors to explaining variability in IoV, ISS, and TGI scores.

The fit of the models was assessed visually. Residuals were checked for normality and homoscedasticity and all the models showed a good enough fit. Correlation matrices showed no problems with multicollinearity.

The full list of R packages and functions used in the analysis is presented in Supplementary File S3.

### 2.7. Ethical approval

The study was approved by Cardiff University School of Medicine Research Ethics Committee (SMREC 20/59) and conducted in accordance with the Declaration of Helsinki. All respondents provided informed consent.

## 3. Results

A total of 711 participants answered the survey at baseline (T1), 383 answered it in the second round (T2) and 295 answered it in the third round (T3), including 35 who had not completed T2. A total of 185 answered it on the fourth round (T4), two of whom had only completed T1 (but not T2 or T3) and 19 who had completed either T2 or T3. A total of 165 participants completed the survey at all time points.

### 3.1. Characteristics of the participants

Table 1 shows the characteristics of the participants for each round of surveys. The average age of participants was around 50 and most participants were women, heterosexual and white. Across the four rounds of the study there was a tendency of the youngest and oldest participants to stop engaging, as well as those from minoritized ethnic backgrounds and those with lowest qualification levels. Over three quarters of the participants had not suffered unemployment or further bereavements at baseline, but toward the end of the study approximately 40% had experienced either at some point since the start of the pandemic. Similarly, approximately 40% of participants reported having medical conditions at baseline, but toward the end of the study, this increased to approximately 60% of participants. Overall, there was no strong change in participants’ demographic characteristics throughout the study. Spiritual/religious beliefs, sexual orientation and region were not considered for analyses due to high levels (over 5%) of missing data for these items.

**Table 1:** Characteristics of the bereaved person.

	T1 (*n* = 711)			T2 (*n* = 383)			T3 (*n* = 295)			T4 (*n* = 185)		
Age	Missing	Mean ± SD [Median]	Min-Max	Missing	Mean ± SD [Median]	Min-Max	Missing	Mean ± SD [Median]	Min-Max	Missing	Mean ± SD [Median]	Min-Max
	8	50 ± 13 [50]	18–90	3	51 ± 13 [53]	21–90	4	51 ± 13 [52]	22–86	3	53 ± 12 [55]	22–84
Gender identity	n	%		n	%		n	%		n	%	
Man	74	10.0%		45	12.0%		32	11.0%		25	14.0%	
Woman	628	89.0%		336	88.0%		261	89.0%		159	86.0%	
Non-binary/Other	5	0.7%		1	0.3%		1	0.3%		0	0.0%	
Didn’t respond/Missing data	2			1			1			1		
Sexual orientation	n	%		n	%		n	%		n	%	
Asexual	1	0.2%		0	0.0%		0	0.0%		0	0.0%	
Bisexual/Bicurious/Pansexual	19	3.1%		6	1.8%		5	1.9%		3	1.8%	
Gay/Lesbian/Queer	28	4.6%		22	6.5%		17	6.4%		9	5.4%	
Straight	564	92.0%		313	92.0%		242	91.3%		154	93.0%	
Unsure	1	0.2%		0	0.0%		1	0.4%		0	0.0%	
Didn’t respond/Missing data	98			42			30			19		
Ethnicity	n	%		n	%		n	%		n	%	
White (total)	676	95.3%		368	96.3%		287	97.6%		180	97.8%	
White British	438	64.8%		241	66.5%		179	62.4%		114	63.3%	
White English	111	16.4%		68	18.5%		51	17.8%		31	17.2%	
White Welsh	41	6.1%		21	5.7%		17	5.9%		13	7.2%	
White Scottish	40	5.9%		19	5.2%		20	7.0%		10	5.6%	
White Northern Irish	22	3.3%		10	2.7%		9	3.1%		5	2.8%	
White Irish	7	1.0%		2	0.5%		3	1.0%		3	1.7%	
Any other white background	17	2.5%		7	1.9%		8	2.8%		4	2.2%	
Minoritized ethnic (total)	33	4.7%		14	3.7%		7	2.4%		4	2.2%	
White and Black Caribbean	11	33.3%		3	21.4%		1	14.3%		1	25.0%	
Black Caribbean	4	12.1%		2	14.3%		1	14.3%		0	0.0%	
White and Black African	2	6.1%		0	0.0%		0	0.0%		0	0.0%	
Arab	1	3.0%		1	7.1%		1	14.3%		0	0.0%	
Bangladeshi	2	6.1%		1	7.1%		1	14.3%		1	25.0%	
Pakistani	1	3.0%		0	0.0%		0	0.0%		0	0.0%	
Indian	4	12.1%		3	21.4%		0	0.0%		0	0.0%	
White and Asian	4	12.1%		3	21.4%		2	28.6%		2	50.0%	
Any other Asian background	1	3.0%		0	0.0%		0	0.0%		0	0.0%	
Any other mixed background	3	9.1%		1	7.1%		1	14.3%		0	0.0%	
Didn’t respond/Missing data	2			1			1			1		
Spiritual/Religious beliefs	n	%		n	%		n	%		n	%	
Buddhism	8	1.2%		4	1.1%		2	0.7%		1	0.6%	
Christianity	244	37.7%		144	40.1%		111	39.6%		76	43.4%	
Hinduism	3	0.5%		1	0.3%		0	0.0%		0	0.0%	
Islamism	5	0.8%		2	0.6%		2	0.7%		2	1.1%	
Judaism	6	0.9%		2	0.6%		3	1.1%		2	1.1%	
Sikhism	2	0.3%		1	0.3%		0	0.0%		0	0.0%	
Other	5	0.8%		3	0.8%		3	1.1%		2	1.1%	
Spiritual but not religious	68	10.5%		43	12.0%		31	11.1%		18	10.3%	
Agnostic*	25	3.9%		9	2.5%		8	2.9%		4	2.3%	
Spiritual or religious (total)	349	53.9%		203	56.5%		155	55.4%		103	58.9%	
No religious beliefs	298	46.1%		150	41.8%		125	44.6%		72	41.1%	
Didn’t respond/Missing data	64			24			15			10		
Highest qualification	n	%		n	%		n	%		n	%	
None/GCSEs	108	15.0%		49	13.0%		35	12.0%		25	14.0%	
A-level/apprenticeship/ONC	132	19.0%		68	18.0%		48	16.0%		24	13.0%	
HND/University degree	468	66.0%		266	69.0%		212	72.0%		136	74.0%	
Didn’t respond/Missing data	3			0			0			0		
Region/Country	n	%		n	%		n	%		n	%	
England (total)	517	72.4%		291	75.9%		216	73.7%		132	71.4%	
East Midlands	39	5.5%		22	5.7%		19	6.4%		12	6.5%	
West Midlands	52	7.3%		27	7.0%		20	6.8%		12	6.5%	
East of England	39	5.5%		25	6.5%		19	6.4%		13	7.0%	
Greater London	68	9.6%		43	11.0%		37	13.0%		24	13.0%	
Yorkshire and the Humber	55	7.7%		31	8.1%		25	8.5%		12	6.5%	
North East	40	5.6%		20	5.2%		12	4.1%		6	3.2%	
North West	95	13.0%		45	12.0%		28	9.5%		15	8.1%	
South East	78	11.0%		46	12.0%		29	9.8%		21	11.4%	
South West	51	7.2%		32	8.4%		27	9.2%		17	9.2%	
Northern Ireland	26	3.7%		12	3.1%		10	3.4%		5	2.7%	
Scotland	53	7.5%		28	7.3%		28	9.5%		17	9.2%	
Wales	63	8.9%		37	9.7%		26	8.8%		20	10.8%	
Didn’t respond/Missing data	52			15			15			11		
Medical conditions	n	%		n	%		n	%		n	%	
Yes (at baseline/cumulative)	279	39.8%		162/192	42.7%/50.7%		113/147	38.7%/50.3%		75/111	41%/60.3%	
No (at baseline/cumulative)	422	60.2%		217/187	57.3%/49.3%		179/145	61.3%/49.7%		108/73	59%/39.7%	
Didn’t respond/Missing data	10			5			3			2/1		
Unemployed during the pandemic?	n	%		n	%		n	%		n	%	
Yes (at baseline/cumulative)	55	7.9%		39/56	10.3%/14.8%		24/75	8.3%/25.8%		14/68	7.7%/37.2%	
No (at baseline/cumulative)	645	92.1%		339/322	89.7%/85.2%		267/216	91.7%/74.2%		169/115	92.3%/62.8%	
Didn’t respond/Missing data	11			6			4			2		
Further bereavements during the study?	n	%		n	%		n		%	n	%	
Yes (at baseline/cumulative)	158	22.5%		80/116	21.2%/30.3%		64/103		22.2%/34.9%	38/81	21.0%/43.8%	
No (at baseline/cumulative)	543	77.5%		297/267	78.8%/69.7%		224/192		77.8%/65.1%	143/104	79%/56.2%	
Didn’t respond/Missing data	10			7/1			7/0			4/0		

All variables were taken at baseline only, except for medical conditions, becoming unemployed and other recent bereavements. *Some participants who reported being agnostic were classified as spiritual/religious while others were classified as having no spiritual beliefs or religion, depending on the answers they provided in the long text box.

### 3.2. Characteristics of the deceased

Table 2 shows the characteristics of the deceased person for each round of surveys. Across the study, days since bereavement ranged from 1 to 812 days. The mean age of the deceased across the four timelines was either 72 or 73 and the median was 74, with a range of less than 1 year (during pregnancy) to 102 years. Over 70% of participants lost either their parent (56%) or their partner (21%); 2.1% experienced the death of their child and 3.2% of their sibling. There was an increase in the percentage of those who lost a partner across the four time-points in the study and a decrease in the percentage of those who lost a grandparent or a family member in the “other” category, suggesting lower retention in the latter. A slight majority of deaths were not due to COVID-19 and over 70% were unexpected by the bereaved respondent. Most deaths occurred in the hospital, followed by at home and in a care home. These trends were very similar across the whole study period.

**Table 2:** Characteristics of the deceased.

	T1 (*n* = 711)			T2 (*n* = 383)			T3 (*n* = 295)			T4 (*n* = 185)		
	Missing	Mean ± SD [Median]	Min-Max	Missing	Mean ± SD [Median]	Min-Max	Missing	Mean ± SD [Median]	Min-Max	Missing	Mean ± SD [Median]	Min-Max
Days since death	4	137 ± 65 [152]	1–279	0	242 ± 28 [234]	145–345	0	408 ± 12 [404]	396–481	0	775 ± 11 [774]	762–812
Age	7	72 ± 16 [74]	<1–102	3	72 ± 17 [74]	<1–102	2	73 ± 16 [74]	<1–100	0	72 ± 16 [74]	<1–100
Relationship of the deceased person to the bereaved	n	%		n	%		n	%		n	%	
Partner	152	21.0%		97	25.0%		78	26.0%		57	31.0%	
Parent	395	56.0%		216	56.0%		169	57.0%		102	55.0%	
Grandparent	54	7.6%		15	3.9%		13	4.4%		2	1.1%	
Sibling	23	3.2%		14	3.7%		8	2.7%		5	2.7%	
Child	15	2.1%		10	2.6%		7	2.4%		6	3.2%	
Other family member	46	6.5%		18	4.7%		12	4.1%		5	2.7%	
Colleague or friend	26	3.7%		13	3.4%		8	2.7%		8	4.3%	
Didn’t respond/Missing data	0			0			0			0		
Cause of death	n	%		n	%		n	%		n	%	
COVID (confirmed or suspected)	311	44.0%		164	43.0%		122	41.0%		79	43.0%	
Non-COVID (total)	399	56.0%		219	57.0%		173	59.0%		106	57.0%	
Cancer	156	39.1%		92	42.0%		75	43.4%		49	46.2%	
Other LLC*	118	29.6%		64	29.2%		51	29.5%		31	29.2%	
Non LLC**	112	28.1%		57	26.0%		45	26.0%		25	23.6%	
Do not know	12	3.0%		6	2.7%		2	1.2%		1	0.9%	
Not specified	1	0.3%		0	0.0%		0	0.0%		0	0.0%	
Didn’t respond/Missing data	1			0			0			0		
Was the death expected?	n	%		n	%		n	%		n	%	
Yes	113	16.0%		65	17.0%		58	20.0%		37	20.0%	
No	552	78.0%		293	77.0%		215	73.0%		137	74.0%	
Do not know	43	6.1%		24	6.3%		21	7.1%		10	5.4%	
Didn’t respond/Missing data	3			1			1					
Place of death	n	%		n	%		n	%		n	%	
In hospital	410	58.0%		208	54.0%		160	54.0%		94	51.0%	
In their home	158	22.0%		92	24.0%		74	25.0%		45	24.0%	
In a hospice	37	5.2%		24	6.3%		15	5.1%		11	5.9%	
In a care home	91	13.0%		53	14.0%		42	14.0%		32	17.0%	
Other	11	1.6%		6	1.6%		4	1.4%		3	1.6%	
Do not know	2	0.3%		0	0.0%		0	0.0%		0	0.0%	
Didn’t respond/Missing data	2			0			0			0		

*LLC, Life-limiting condition, for example, heart disease, COPD, dementia. **Examples of non-LLC are stroke, heart attack, accident, suicide.

### 3.3. Characteristics of the experience of bereavement

Table 3 summarizes the characteristics of the experience of bereavement in terms of pandemic-related challenges before or after the death of a loved one and the support received by healthcare professionals immediately following the death. Most participants reported having had restricted funeral arrangements and limited contact with close relatives or friends (over 90% and over 80% across all time points, respectively), while a smaller majority reported a sense of isolation and loneliness (66.7% at baseline, varying by a maximum of 5.7 percentual points across all time points). Smaller majorities also reported being unable to say goodbye as they liked (63.9% at baseline, varying by a maximum of 2.9 percentual points across time points) and having limited contact with the deceased in the last days of their lives (57.8% at baseline, varying by a maximum of 1.2 percentual points across time points), while approximately half of respondents reported being unable to visit the deceased in the last days of their lives (54.3% at baseline, varying by a maximum of 8.2 percentual points across time points). Accordingly, a greater proportion of people reported facing all three challenges related to social isolation (60%, at baseline, varying by a maximum of 7 percentual points across time points) compared to the proportion of people who reported facing all three challenges related to contact prior to death (35%, at baseline, varying by a maximum of 2.6 percentual points across time points). Less than 2% of people reported not facing any of the COVID-19 related challenges and around a quarter of respondents reported facing all six challenges. Approximately 20% reported facing three, four or five of the challenges. Over half of the participants felt little or no support by healthcare professionals and only 20% reported feeling very well supported. Changes in these proportions across time points do not suggest major bias in sample retention regarding experience of bereavement.

**Table 3:** Characteristics of the experience of bereavement regarding COVID-19 restrictions and perceived level of support offered by healthcare professionals.

	T1 (*n* = 711)		T2 (*n* = 383)		T3 (*n* = 295)		T4 (*n* = 185)	
Contact prior to death	n (yes)	%	n (yes)	%	n (yes)	%	n (yes)	%
Unable to visit them prior to their death	386	54.3%	188	49.0%	136	46.1%	87	47.0%
Unable to say goodbye as I would have liked	454	63.9%	238	62.1%	180	61.0%	114	61.6%
Limited contact with them in last days of their life	411	57.8%	222	58.0%	174	59.0%	107	57.8%
Social isolation	n (yes)	%	n (yes)	%	n (yes)	%	n (yes)	%
Restricted funeral arrangements	664	93.4%	363	94.8%	280	94.9%	176	95.1%
Limited contact with other close relatives or friends	574	80.7%	318	83.0%	253	85.8%	157	84.9%
Sense isolation and loneliness	474	66.7%	267	69.7%	200	67.8%	134	72.4%
Number of negative experiences related to contact prior to death	n	%	n	%	n	%	n	%
0	147	21.0%	88	23.0%	74	25.0%	44	23.8%
1	128	18.0%	69	18.0%	50	17.0%	34	18.4%
2	185	26.0%	99	26.0%	73	25.0%	47	25.4%
3	251	35.0%	127	33.0%	98	33.0%	60	32.4%
Number of negative experiences related to social isolation	n	%	n	%	n	%	n (yes)	%
0	26	3.7%	11	2.9%	7	2.4%	3	1.6%
1	87	12.0%	44	11.0%	33	11.0%	21	11.4%
2	169	24.0%	80	21.0%	65	22.0%	37	20.0%
3	429	60.0%	248	65.0%	190	64.0%	124	67.0%
Total number of negative experiences	n	%	n	%	n	%	n	%
0	9	1.3%	5	1.3%	4	1.4%	1	0.5%
1	38	5.3%	17	4.4%	12	4.1%	7	3.8%
2	68	9.6%	40	10.0%	32	11.0%	18	9.7%
3	127	18.0%	68	18.0%	56	19.0%	38	20.5%
4	127	18.0%	68	18.0%	49	17.0%	32	17.3%
5	152	21.0%	87	23.0%	69	23.0%	44	23.8%
6	190	27.0%	98	26.0%	73	25.0%	45	24.3%
Felt supported by healthcare professionals	n	%	n	%	n	%	n	%
Not at all supported	252	35.0%	138	36.0%	101	34.0%	68	36.8%
A little bit supported	139	20.0%	70	18.0%	62	21.0%	44	23.8%
Fairly well supported	105	15.0%	61	16.0%	44	15.0%	23	12.4%
Very well supported	95	13.0%	61	16.0%	50	17.0%	30	16.2%
Not relevant (not next of kin)	120	17.0%	53	14.0%	38	13.0%	20	10.8%
Didn’t respond/Missing data	0		0		0		0	

### 3.4. Grief and social support outcomes

Table 4 summarizes the participants’ outcome measures across the study.

**Table 4:** Summary of participants’ grief and support outcomes across the study.

	T1 (*n* = 711)			T2 (*n* = 383)			T3 (*n* = 295)			T4 (*n* = 185)		
				Missing	Mean ± SD [Median]	Min-Max	Missing	Mean ± SD [Median]	Min-Max	Missing	Mean ± SD [Median]	Min-Max
ISS (1–5)				2	3.3 ± 0.9 [3.2]	1–5	0	3.3 ± 0.9 [3.2]	1–5	0	3.3 ± 0.9 [3.2]	1–5
TGI (PGD score) (18–90)				1	51.5 ± 14.6 [52]	22–90	0	48.5 ± 14.7 [48]	18–89	0	44.7 ± 15.1 [43]	20–90
PGD Diagnosis (≥54)				n	%		n	%		n	%	
Yes				167	43.7%		102	34.6%		53	28.6%	
No				215	56.3%		193	65.4%		132	71.4%	
Didn’t respond/Missing data				1			0			0		
	Missing	Mean ± SD [Median]	Min-Max	Missing	Mean ± SD [Median]	Min-Max	Missing	Mean ± SD [Median]	Min-Max	Missing	Mean ± SD [Median]	Min-Max
AAG (IoV score) (0–34)	13	20.4 ± 4.8 [21]	4–34	5	19.6 ± 4.9 [20]	6–31	0	18.7 ± 4.4 [19]	5–33	0	18.3 ± 4.7 [18]	6–29
IoV risk	n	%		n	%		n	%		n	%	
Low (0–20)	338	48.4%		218	57.7%		200	67.8%		125	67.6%	
High (21–23)	163	23.4%		71	18.8%		58	19.7%		35	18.9%	
Extreme (≥ 24)	197	28.2%		89	23.5%		37	12.5%		25	13.5%	
Didn’t respond/Missing data	13			5			0			0		

TGI: Mean TGI (PGD) score at T2 was 51.5. This decreased steadily across survey time points, dropping to 48.5 at T3 and 44.7 at T4. At T2 43.7% met the threshold for indicated PGD (≥54), dropping to 34.6% at T3 (c.13 months post-bereavement) and 28.6% at final follow up, c. 25 months post-bereavement.

AAG: Mean IoV (grief vulnerability) score at baseline was 20.4, decreasing slightly but steadily to 18.7 at T3 and 18.3 at 24. At baseline 48.4% exhibited low levels of vulnerability (i.e., 0 ≤ IoV ≤ 20); 23.4% exhibited high levels (i.e., 21 ≤ IoV ≤ 23), and 28.2% exhibited severe levels (i.e., IoV ≥ 24). By T4 67% exhibited low levels of vulnerability, 18.9% exhibited high levels and 13.5% demonstrated severe levels.

ISS: Social support scores were stable and did not change across survey time points. Mean ISS score at T2,T3,T4 was 3.3 (see Table 4). This could be interpreted as feeling “fairly well supported,” with a score of 5 meaning “very well” and a score of 1 meaning “not at all.”

### 3.5. Factors associated with levels of prolonged grief symptoms, social support and vulnerability in grief

**Figure 1:**
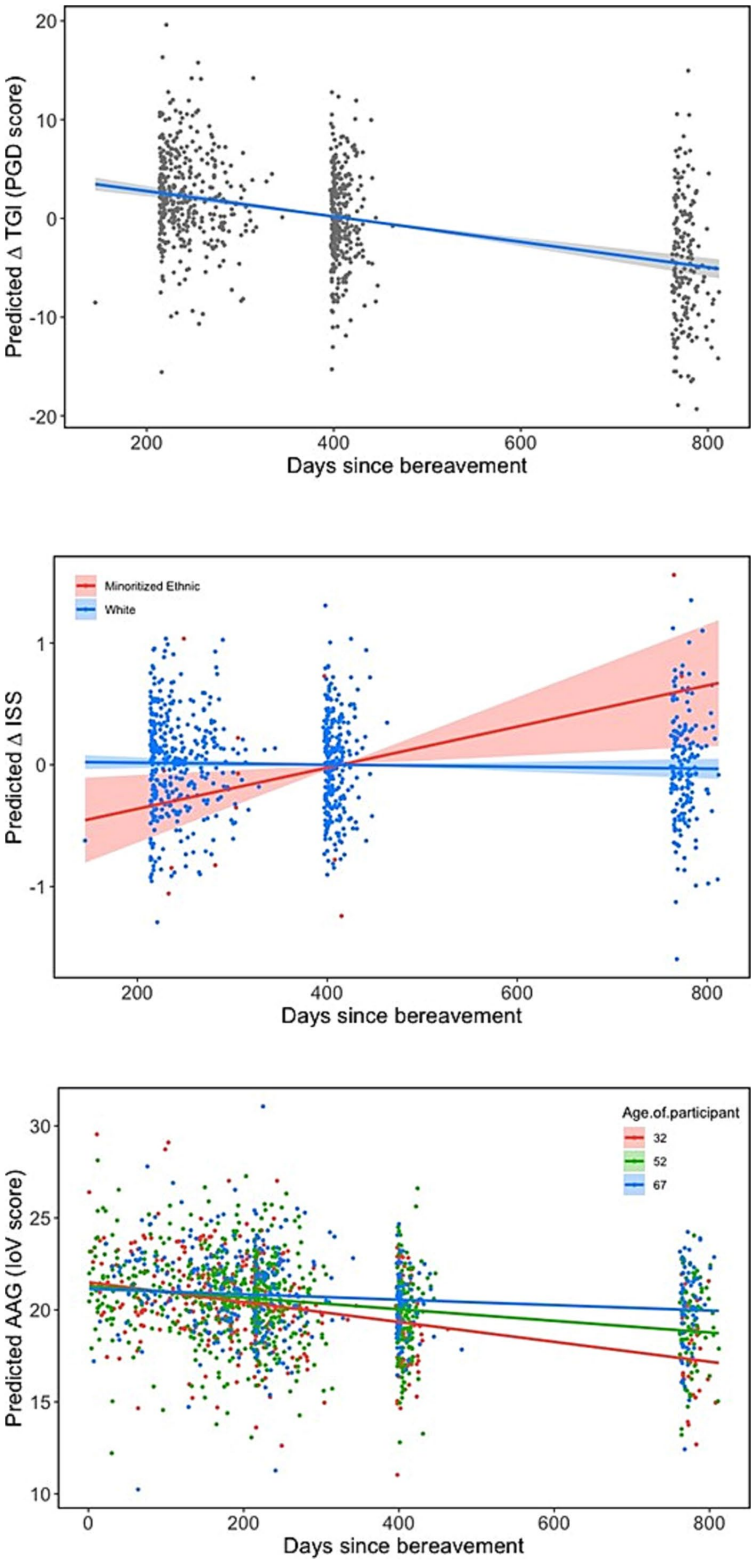
Predicted temporal patterns of TGI, ISS, and loV scores regarding days since bereavement. ISS and lov score showed significant interactions between days since bereavement and, respectively, ethnicity and age of participant, which are depicted here. Note that TGI scores and ISS are plotted as contrasts, showing the predicted average change in TGI score and ISS across all groups of participants, while IoV is depicted as predicted scores for the first, second and third quartiles of participants’ ages while keeping all other variables constant (set for the group with larger sample size); predicted scores were plotted instead of contrasts because the interaction was more visually easily interpreted this way.

**Figure 2:**
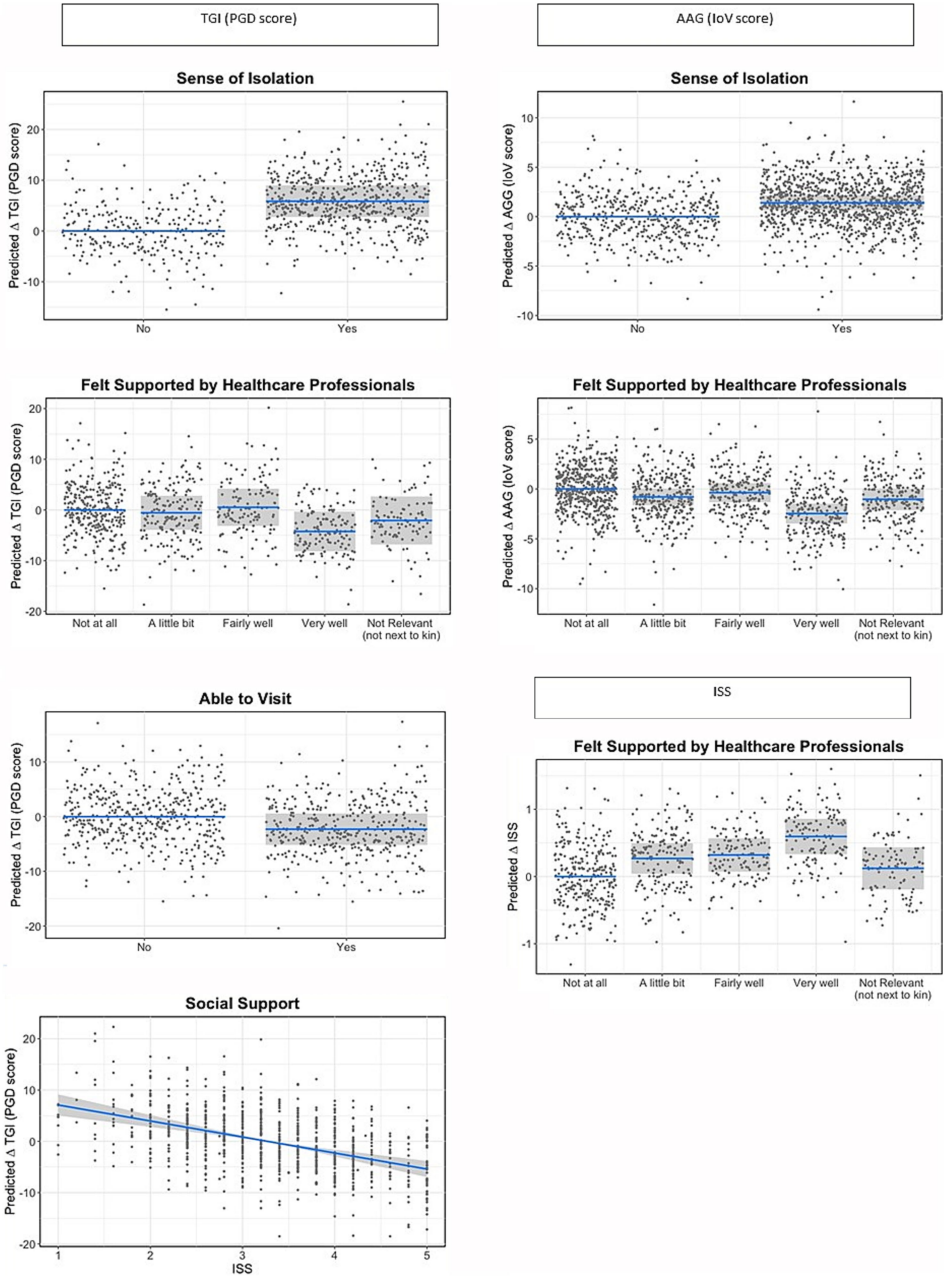
Predicted changes (statistical contrasts) in TGI, ISS, and loV scores across different experiences of bereavement identified in the statistical models as having the strongest effect sizes for each outcome. Shaded areas represent 95% confidence intervals for the change in TGI, ISS, or lov score in relation to the reference category. Note that TGI scores range from 18 to 90, lov from 0 to 34 and ISS from 1 to 5, and hence the same change in TGI and lov represents an approximate twofold change in magnitude for lov compared to TGI, while a change in ISS represents an approximate sevenfold change in magnitude compared to lov and 14-fold change in magnitude compared to TGI.

**Figure 3:**
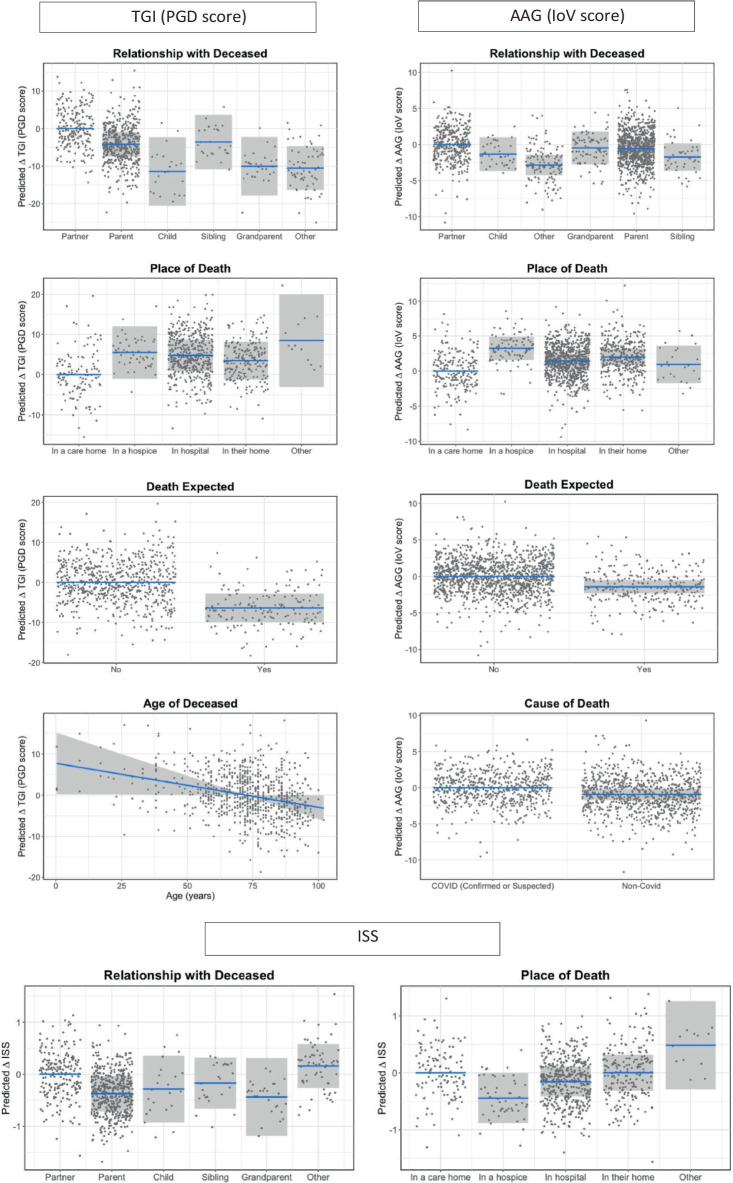
Predicted changes (statistical contrasts) in TGI, ISS, and IoV scores in relation to characteristics of the deceased and circumstances of death identified in the statistical models as having the strongest effect sizes for each outcome. Shaded areas represent 95% confidence intervals for the change in TGI, ISS or IoV score in relation to the reference category. Note that TGI scores range from 18 to 90, IoV from 0 to 34 and ISS from 1 to 5, and hence the same change in TGI and IoV represents an approximate twofold change in magnitude for IoV compared to TGI, while a change in ISS represents an approximate sevenfold change in magnitude compared to IoV and 14-fold change in magnitude compared to TGI.

**Figure 4:**
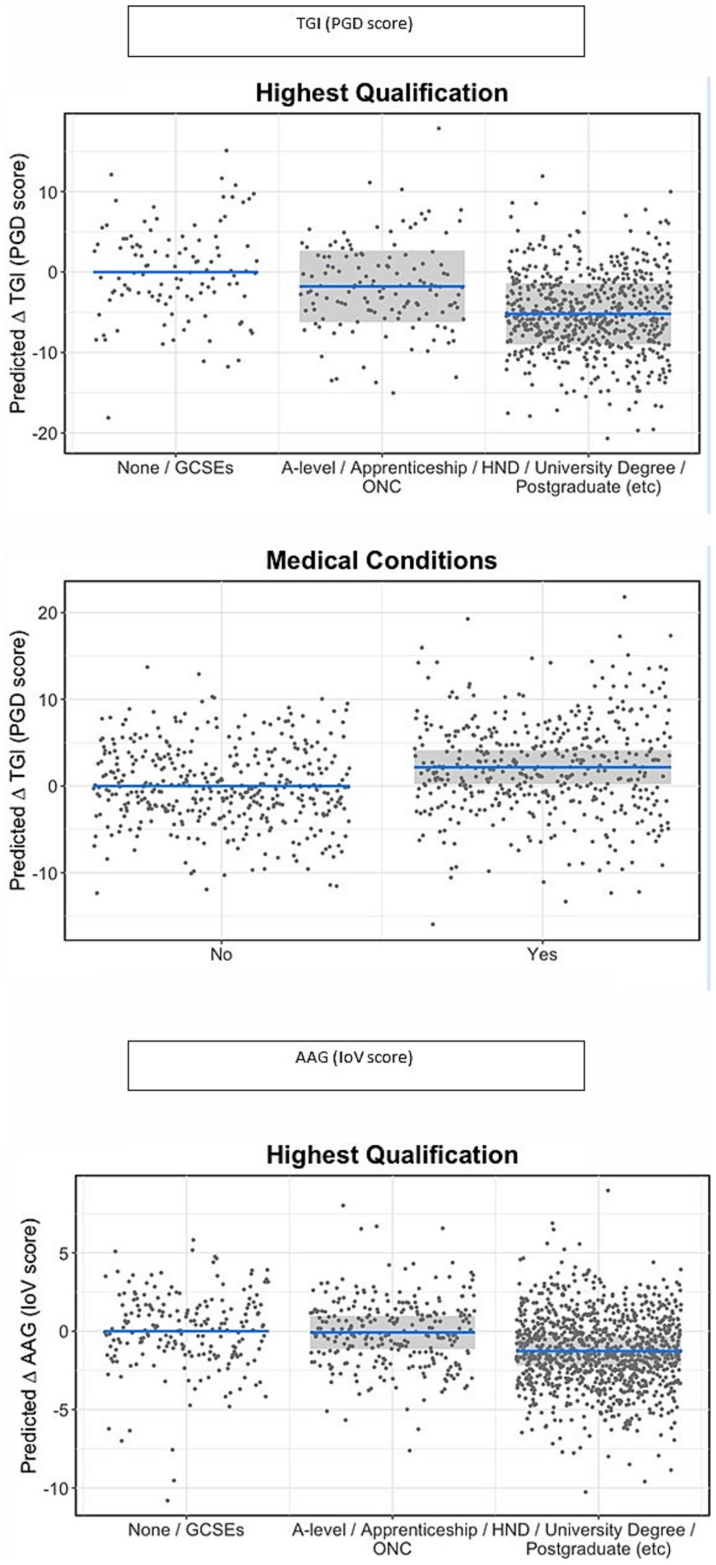
Predicted changes (statistical contrasts) in TGI and loV scores in relation to characteristics of the participants identified in the statistical models as having the strongest effect sizes for each outcome. Shaded areas represent 95% confidence intervals for the change in TGI or lov score in relation to the reference category. Note that TGI scores range from 18 to 90 and lov from 0 to 34, hence the same change in TGI and loV represents an approximate twofold change in magnitude for lov compared to TGI.

Table 5 shows the effect sizes for each individual variable from the single models on TGI (PGD symptoms), ISS and IoV and the marginal R^2^ values for the models containing each set of variables. Table 6 shows the model outputs for the full models containing all groups of variables in combination to assess the effect of the experience of bereavement in each of the outcome variables, while controlling for the characteristics of the participant/bereaved and the characteristics of the deceased; Figures 1–4 show the model predictions for TGI (PGD symptoms), ISS and IoV for variables that had the largest effect sizes.

**Table 5:** Results of the mixed models with participant ID as random term, days since bereavement as co-variate and each of the predictors individually.

	TGI (PGD score) (18–90)				ISS (1–5)				AAG (IoV score) (0–34)			
	Coeff.	SE	DF	Partial R	Coeff.	SE	DF	Partial R	Coeff.	SE	DF	Partial R
Age of participant	0.038	0.056	408	0.03	0.0008	0.003	406	−0.02	0.011	0.013	680	0.03
	Diff	SE	DF	Cohen’s *d*	Diff	SE	DF	Cohen’s *d*	Diff	SE	DF	Cohen’s *d*
Gender identity												
Man—Woman	−4.34	2.24	408	−0.648	0.19	0.13	401	0.351	−0.27	0.55	657	−0.096
Ethnicity												
White—Minoritized ethnic	7.80	3.94	432	1.160	−0.53	0.23	441	−0.977	0.71	0.80	753	0.249
Highest qualification												
A-level/apprenticeship/ONC—None/GCSEs	−3.16	2.55	419	−0.478	0.15	0.15	421	0.286	−0.55	0.58	703	−0.195
HND/University degree/Postgraduate (etc.)—None/GCSEs	−8.62	2.12	414	−1.307	0.13	0.13	412	0.249	−2.33	0.48	699	−0.823
HND/University Degree/Postgraduate (etc.)—A-level/apprenticeship/ONC	−5.45	1.84	423	−0.829	−0.02	0.11	428	−0.037	−1.78	0.43	692	−0.628
Medical conditions												
Yes—No	2.53	1.09	829	0.375	−0.12	0.07	714	−0.216	0.71	0.24	1,531	0.150
Unemployed during the pandemic												
Yes—No	−0.72	1.09	786	−0.107	0.06	0.08	845	0.110	−0.61	0.33	1,496	−0.215
Further bereavements during the study												
Yes—No	−0.64	1.08	858	−0.095	<−0.0001	0.07	790	−0.0001	−0.24	0.28	1,527	−0.0854
Marginal R2 (95%CI)	0.086 (0.061–0.134)				0.023 (0.019–0.064)				0.063 (0.047–0.094)			
	Coeff.	SE	DF	Partial R	Coeff.	SE	DF	Partial R	Coeff.	SE	DF	Partial R
Days since death (scaled)	−2.538	0.262	471	−0.41	−0.001	0.021	499	−0.02	−0.601	0.082	1,009	−0.23
Age of deceased	−0.171	0.043	408	−0.2	−0.005	0.003	405	−0.1	−0.047	0.01	668	−0.17
	Diff	SE	DF	Cohen’s *d*	Diff	SE	DF	Cohen’s *d*	Diff	SE	DF	Cohen’s *d*
Relationship of the deceased person to the bereaved (reference = Partner)												
Parent	−6.79	1.61	405	−1.011	−0.31	0.10	398	−0.581	−1.92	0.40	646	−0.676
Grandparent	−17.60	3.54	428	−2.623	−0.30	0.22	438	−0.561	−2.86	0.69	759	−1.010
Sibling	−4.23	4.05	415	−0.630	−0.42	0.24	420	−0.771	−1.30	0.97	678	−0.459
Child	−4.29	4.52	401	−0.639	−0.18	0.27	390	−0.337	0.35	1.13	640	0.123
Other family member or a colleague or friend	−15.70	2.75	415	−2.339	0.12	0.17	413	0.220	−4.42	0.63	699	−1.560
Cause of death												
Non-COVID—COVID	−6.01	1.40	416	−0.897	0.18	0.08	414	0.332	−1.66	0.33	683	−0.587
Was the death expected?												
Yes—No	−9.05	1.79	412	−1.350	0.21	0.11	407	0.391	−2.06	0.44	659	−0.728
Place of death (reference = in a care home)												
In hospital	9.12	2.10	404	1.359	−0.09	0.13	395	−0.170	2.30	0.51	663	0.811
In their home	2.87	2.34	405	0.429	0.10	0.14	397	0.194	1.54	0.57	657	0.542
In a hospice	6.58	3.43	409	0.982	−0.15	0.21	402	−0.284	2.67	0.85	663	0.942
Other	9.54	6.03	408	1.423	0.47	0.36	400	0.868	1.56	1.39	685	0.551
Marginal R2 (95%CI)	0.210 (0.174–0.266)				0.056 (0.042–0.103)				0.145 (0.120–0.183)			
	Diff	SE	DF	Cohen’s *d*	Diff	SE	DF	Cohen’s *d*	Diff	SE	DF	Cohen’s *d*
Contact prior to death (Yes—No)												
Unable to visit them prior to their death	−0.52	1.41	417	−0.078	−0.18	0.08	414	−0.335	0.13	0.33	678	0.045
Unable to say goodbye as I would have liked	1.23	1.45	416	0.183	−0.10	0.09	414	−0.194	0.55	0.35	682	0.194
Limited contact with them in last days of their life	1.57	1.43	417	0.234	−0.01	0.08	414	−0.026	0.08	0.34	682	0.029
Social isolation (Yes—No)												
Restricted funeral arrangements	3.66	3.23	413	0.545	−0.32	0.19	408	−0.590	1.52	0.69	719	0.537
Limited contact with other close relatives or friends	5.60	1.91	419	0.834	−0.24	0.11	420	−0.452	0.98	0.43	709	0.346
Sense isolation and loneliness	9.42	1.48	411	1.400	−0.16	0.09	406	−0.305	2.30	0.35	691	0.812
Feel supported (reference = Not at all supported)												
A little bit supported	−1.55	1.95	408	−0.231	0.29	0.11	400	0.534	−0.92	0.46	675	−0.326
Fairly well supported	−0.71	2.07	421	−0.107	0.35	0.12	424	0.645	−0.91	0.50	675	−0.322
Very well supported	−7.31	2.08	410	−1.091	0.64	0.12	404	1.183	−2.82	0.51	651	−0.999
Not relevant (not next of kin)	−8.72	2.23	416	−1.301	0.32	0.13	417	0.594	−2.54	0.49	711	−0.898
	Coeff.	SE	DF	Partial R	Coeff.	SE	DF	Partial R	Coeff.	SE	DF	Partial R
Total number of negative experiences	1.439	0.453	411	0.15	−0.065	0.027	404	−0.12	0.363	0.106	691	0.13
ISS	−3.194	0.473	790	−0.23								
Marginal R2 (95%CI)*	0.171 (0.138–0.225)				0.070 (0.051–0.117)				0.101 (0.08–0.135)			

The table is split by groups of variables: characteristics of the participants in yellow; characteristics of the deceased in salmon and characteristics of the experience of the bereavement in blue. Marginal R-squared are presented for the models containing each group of variables. Partial R and the Cohen’s *d*, the intensity of the shading reflects the strength of the effect.

*Model do not converge with all variables so ran it without number of negative experiences, as the variable with smallest effect.

**Table 6:** Outcomes of the General Linear Mixed models for TGI score, ISS, and AAG score with participant ID as random term, days since bereavement as covariate and all the predictors in combination (excluding those with very small effects for each given model that were necessary to remove for the model to fit appropriately—further bereavements during the study and total number of negative experiences were included in the starting models but were removed from all the models due to small effects).

	TGI (PGD score)			ISS			AAG (IoV score)		
Predictors [Reference category]	Estimates	95% CI	*p*	Estimates	95% CI	*p*	Estimates	95% CI	*p*
(Intercept)	68.31	54.26 to 82.37	**<0.001**	3.91	2.95 to 4.87	**<0.001**	22.97	20.07 to 25.87	**<0.001**
Days (scaled)	−2.69	−3.21 to −2.17	**<0.001**	0.35	0.08 to 0.63	**0.011**	−1.88	−2.57 to −1.19	**<0.001**
Gender—Male [Female]	−1.86	−5.84 to 2.13	0.361	0.14	−0.13 to 0.42	0.298	*	*	*
Ethnicity—White [Minoritized Ethnic]	4.9	−2.40 to 12.21	0.188	−0.18	−0.70 to 0.33	0.487	*	*	*
Medical conditions—Yes [No]	2.17	0.21 to 4.13	**0.03**	−0.12	−0.26 to 0.02	0.102	*	*	*
Unemployed during the pandemic—Yes [No]	*	*	*	0.01	−0.14 to 0.17	0.854	*	*	*
Highest qualification—A-level/apprenticeship/ONC [No qualifications]	−1.79	−6.25 to 2.67	0.432	0.07	−0.24 to 0.37	0.663	−0.08	−1.14 to 0.98	0.886
Highest qualification—HND/University Degree/Postgraduate (etc.) [No qualifications]	−5.19	−9.01 to −1.38	**0.008**	0.03	−0.24 to 0.29	0.851	−1.27	−2.19 to −0.35	0.007
Age of participant				−0.01	−0.02 to 0.01	0.372	0.02	−0.02 to 0.06	0.27
Relationship—Parent [Partner]	−4.18	−7.49 to −0.87	**0.013**	−0.38	−0.72 to −0.03	**0.031**	−0.57	−1.75 to 0.60	0.34
Relationship—Sibling [Partner]	−3.58	−10.87 to 3.70	0.335	−0.17	−0.66 to 0.32	0.499	−1.73	−3.62 to 0.16	0.072
Relationship—Child [Partner]	−11.42	−20.58 to −2.25	**0.015**	−0.29	−0.93 to 0.36	0.383	−1.32	−3.66 to 1.01	0.267
Relationship—Grandparent [Partner]	−10.03	−17.84 to −2.22	**0.012**	−0.44	−1.19 to 0.31	0.252	−0.47	−2.74 to 1.80	0.683
Relationship—Other [Partner]	−10.52	−16.37 to −4.68	**<0.001**	0.16	−0.26 to 0.58	0.465	−2.84	−4.26 to −1.42	**<0.001**
Death expected—Yes [No]	−6.39	−9.96 to −2.81	**<0.001**	0.04	−0.20 to 0.28	0.737	−1.42	−2.32 to −0.51	**0.002**
Place of death—In a hospice [Care home]	5.5	−1.02 to 12.02	0.098	−0.44	−0.88 to −0.00	**0.047**	3.24	1.58 to 4.89	**<0.001**
Place of death—In hospital [Care home]	4.77	0.91 to 8.64	**0.016**	−0.16	−0.42 to 0.10	0.241	1.42	0.45 to 2.39	**0.004**
Place of death—In their home [Care home]	3.45	−1.23 to 8.14	0.148	0	−0.31 to 0.32	0.991	2	0.82 to 3.18	**0.001**
Place of death—Other [Care home]	8.46	−3.11 to 20.04	0.152	0.48	−0.29 to 1.26	0.221	0.95	−1.73 to 3.62	0.487
Cause of death—Non-Covid [Covid]	−1.32	−4.42 to 1.78	0.405	0	−0.21 to 0.21	0.978	−0.93	−1.70 to −0.17	**0.016**
									
Age of deceased person	−0.11	−0.21 to −0.00	**0.043**	0	−0.01 to 0.01	0.769	−0.05	−0.08 to −0.01	**0.011**
Limited contact last days—Yes [No]	1.44	−1.36 to 4.25	0.313	0.14	−0.06 to 0.33	0.17	−0.24	−0.95 to 0.47	0.506
Restricted funeral—Yes [No]	−1.7	−7.54 to 4.14	0.567	−0.18	−0.57 to 0.22	0.385	−0.02	−1.37 to 1.33	0.974
Sense of Isolation—Yes [No]	5.86	2.75 to 8.97	**<0.001**	−0.05	−0.26 to 0.17	0.664	1.4	0.64 to 2.16	**<0.001**
Limited contact relatives—Yes [No]	0.46	−3.26 to 4.17	0.809	−0.11	−0.36 to 0.14	0.372	−0.18	−1.06 to 0.69	0.681
Unable to visit—Yes [No]	2.29	−0.58 to 5.17	0.118	0.13	−0.06 to 0.33	0.181	0.23	−0.49 to 0.95	0.528
Unable to say goodbye—Yes [No]	1.43	−1.84 to 4.71	0.39	−0.14	−0.36 to 0.08	0.221	−0.03	−0.84 to 0.78	0.944
Feel supported—A little bit supported [Not at all supported]	−0.55	−3.92 to 2.82	0.749	0.27	0.04 to 0.50	**0.02**	−0.8	−1.64 to 0.03	0.06
Feel supported—Fairly well supported [Not at all supported]	0.54	−3.14 to 4.22	0.774	0.32	0.07 to 0.57	**0.011**	−0.37	−1.33 to 0.58	0.443
Feel supported—Very well supported [Not at all supported]	−4.23	−8.15 to −0.31	**0.034**	0.6	0.33 to 0.86	**<0.001**	−2.46	−3.46 to −1.47	**<0.001**
Feel supported—Not relevant to my situation [Not at all supported]	−2.06	−6.80 to 2.67	0.392	0.12	−0.19 to 0.44	0.446	−1.04	−2.12 to 0.05	0.062
ISS	−3.12	−4.04 to −2.21	**<0.001**						
Days (scaled) × Ethnicity grouped [White]				−0.37	−0.64 to −0.10	**0.008**			
Days (scaled) × Age of participant							0.02	0.01 to 0.04	**<0.001**
**Random effects**									
σ^2^	43.31			0.28			7.91		
τ_00 respondents_	119.58			0.48			10.7		
ICC	0.73			0.63			0.57		
N _respondents_	411			402			672		
Sample size	842			821			1,510		
Marginal R^2^/Conditional R^2^	0.311/0.817			0.119/0.674			0.206/0.662		

*Variables with very small effect sizes that were removed from the final model.

The *p* values highlighted in bold are less than 0.05 (i.e. statistically significant).

Across all the analyses, characteristics of the deceased were generally and consistently the best predictors of all three indices: TGI (PGD symptoms), ISS and IoV. For TGI and IoV, relationship with deceased, followed by place of death, showed the largest effect sizes across all variables, while for ISS, the largest effects were from feeling supported by health care professionals following the death and ethnicity, only then followed by place of death and relationship with deceased. Ethnicity also showed a large effect on TGI, with white respondents showing worse grief outcomes compared to minoritized ethnic respondents, but not on IoV. Qualifications showed large effects for TGI and IoV, with those from lower education levels showing worse grief outcomes, but not for ISS. All items related to contact prior to death showed small effects for all three indices, while items for social isolation showed large and very large effects for TGI and IoV; specifically sense of isolation and loneliness had a very large effect on TGI and a large effect on IoV, while restricted funeral arrangements had a large effect on TGI but a medium effect on IoV. Days since death had a larger effect on TGI than IoV or ISS; although the effect of the slope is not very large even for TGI, it represents a considerable change in TGI scores over an extended period of time (e.g., an approximate reduction of 4 scale points in TGI for each 5 months).

### 3.6. Effect of time

Figure 1 shows trends of TGI, ISS, and IoV in relation to time since bereavement. There was a general tendency for TGI and IoV to improve with time, which was more noticeable for TGI than for IoV. Furthermore, there was an interaction between days since bereavement and age of participant for IoV, with younger participants improving more through time compared to older participants. The relationship between days since bereavement and ISS is less strong with a slight tendency for ISS to improve with time, although this is mainly driven by those from minoritized ethnic groups, with white participants showing no change in ISS through time. Although only the linear trend was fitted for simplicity, visual analysis showed that the sharpest decline in grief scores seem to occur between 6 months to a year since bereavement.

**Figure 5:**
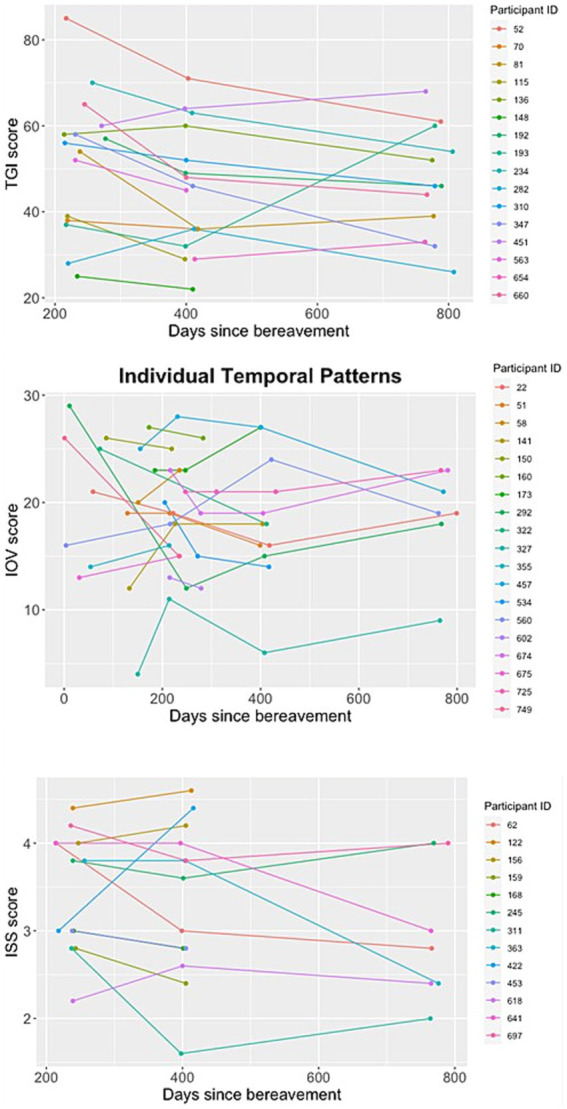
Temporal patterns of TGI, ISS and IoV scores regarding days since bereavement for different randomly generated selections of study participants.

These were general patterns averaged across participants, but it was noticeable that for all indices of bereavement different participants would show different patterns, with some improving through time, some worsening through time, some showing oscillations but no real trend and some showing no change (Figure 5).

### 3.7. Effect of experience of bereavement

The results from the full models examining the effects of the experiences of bereavement while controlling for characteristics of the participant and the bereaved, showed that sense of isolation and feeling supported by healthcare professionals were the most important experiences in predicting TGI and IoV scores, while only the latter was an important predictor of ISS. Those who felt very well supported by healthcare professionals showed better grief and support outcomes compared to all other groups. ISS score was also an important predictor of TGI; in the same model but without ISS, feeling supported by healthcare professionals showed a much larger effect on TGI than when ISS was included, which suggests that ISS has a mediating effect between feeling supported and TGI levels, while sense of isolation has both a direct and indirect effect on TGI. These patterns are depicted in Figure 2.

Despite representing a relatively modest effect, being unable to visit a loved one prior to death had an increased effect on TGI once all other factors were accounted for (an estimated average difference of 2.3 scale points in TGI level). Restricted funeral arrangements showed a much smaller effect for IoV when other variables were controlled for than in the single model. Number of negative experiences during bereavement showed very low effect sizes across all three indices, once other variables were accounted for.

### 3.8. Effect of the characteristics of the participant and the deceased

Place of death and relationship with the deceased remained the strongest predictors of grief and support outcomes, although the effect of relationship with the deceased was generally smaller when other factors were accounted for, most noticeably for IoV. Specifically, the estimated differences in IoV between losing a partner or a parent or grandparent were much smaller than when the variable was considered in the single model. Those bereaved of a partner showed higher TGI scores and slightly higher IoV scores compared to other groups. Losing a child showed small differences in TGI and IoV scores compared to losing a partner when looked at in isolation, but when other factors were accounted for, those bereaved of a child showed smaller TGI and IoV scores than all other groups. However, confidence intervals for these differences are wide (Figure 3) and these contradictory results seem more likely to be due to the small sample size for those bereaved of a child. Those bereaved of a partner and those bereaved of a distant relative or friend showed greater ISS scores compared to all other groups.

There were generally worse outcomes for those who had a loved one dying in a hospice and best for those who had a loved one dying in a care home, with TGI scores also high for those on the “other” category. ISS scores were lowest for deaths in hospices and highest for the “other” category.

An unexpected death had a negative effect on both TGI and IoV, while cause of death only had a meaningful influence on IoV—those who lost someone to COVID-19 had slightly worse outcomes, while age of deceased only had a meaningful influence on TGI, with worse outcomes for bereavements of younger people. Although only the linear trend was fitted for simplicity, visual analysis of the relationship between age of deceased and TGI showed a sharper decrease from the age of 70 years (this was also the case for IoV and ISS scores also showed a slightly more pronounced decrease from this point). These patterns are shown in Figure 3.

Qualifications also showed important effects for TGI and IoV, with those with higher education levels showing better outcomes. Having medical conditions showed a negative effect for TGI. Ethnicity showed an estimated average difference of around 5 scale points in TGI level (with higher TGI scores for white group), but due to the small sample size and large variability in the minoritized ethnic group and potentially its relatively high association with ISS, this variable has an overall small effect size on TGI when controlling for other variables. These patterns are shown in Figure 4.

Age of participant, further bereavements during the study, and whether people were unemployed during the pandemic showed the lowest effect sizes across all three indices, once other variables were accounted for.

## 4. Discussion

This analysis represents the only published longitudinal examination of COVID-19 pandemic grief outcomes that we are aware of, with a focus on symptoms of PGD as our primary outcome. In a sample of people bereaved during the first 9 months of the COVID-19 pandemic in the UK, we found decreasing but relatively high levels of indicated PGD at c. 8 (44%), 13 (35%), and 25 (29%) months post-bereavement. Factors most strongly associated with levels of PGD symptoms were those relating to the person who died, in terms of their relationship to the participant, where they died and whether the death was expected. Large effects relating to support were also observed, specifically isolation and loneliness around the time of bereavement and perceived social support over time, with support from healthcare professionals immediately following the death also a factor. Level of education and existence of medical conditions were the main participant characteristics found to have an effect. These findings have important implications for bereavement policy, provision and practice in the current COVID-recovery and post-pandemic period (e.g., strengthening of specialist and social support) and in preparedness for future pandemics and mass bereavement events (e.g., infection control measures and rapid support responses).

### 4.1. Grief levels and the effects of time

We found relatively high levels of indicated PGD and grief vulnerability (IoV) overall, and across time. As in other studies, time since death was negatively associated with overall levels of PGD symptoms (8, 34), and to a lesser extent levels of grief vulnerability (IoV), with a pattern of improvement and “normal” grief trajectories for many (5, 6). However, there were also patterns of worsening grief and grief which remained relatively static over time (52) and the proportions of people meeting the threshold for indicative PGD remained higher than would be expected in non-pandemic times, with around 36% at c. 13 months bereaved and around 29% at c. 25 months. Public health models of bereavement (9, 11) suggest that in non-pandemic times, around 10% of bereaved people are at high risk of PGD and may need professional mental health support, and a further 30% are at moderate risk and may need some additional support, e.g., via peer support groups. These estimates were confirmed in a 2015 Australian survey, which in a sample of people on average 14 months bereaved, identified 6.4% at high-risk of PGD, 35.2% at moderate risk and the remaining 58.4% at low risk (9). Although our sample is limited by its self-selecting design and is not representative, our findings would nonetheless appear to support predictions that grief disorder prevalence would rise because of the pandemic (2–4), providing longer-term evidence that is consistent with the results of earlier cross-sectional studies with more recently bereaved populations (14–20).

We now summarize the factors associated with poorer grief outcomes and consider which of these factors might explain the apparently higher levels of indicated PGD that we observed in this pandemic-bereaved cohort.

### 4.2. The person who died

We found that, over time, relationship to the deceased continued to be strongly associated with symptoms of PGD and grief vulnerability, as in our baseline analysis (23). People whose partner, child or sibling died showed higher levels of grief compared with bereavements of more distant relatives/friends, although group sizes for child and sibling loss were very small. These effects became relatively less important once other factors were controlled for, especially in relation to grief vulnerability (IoV), and were influenced by perceived social support in the PGD symptoms (TGI) model. Specifically, our results suggest that people who lost parents and siblings experienced poorer social support, which might have contributed to their relatively worse levels of PGD symptoms, while the better support experienced by bereaved spouses/partners could have buffered the effects of their loss, despite their overall higher grief levels. These varying levels of support also demonstrate greater perceived lack of understanding and empathy within social networks in relation to deaths of siblings, parents and grandparents, or greater reluctance to seek or ask for help among those experiencing these types of bereavement, as also indicated in our qualitative findings (21).

Age of the deceased had a small but significant effect on symptoms of PGD [although not grief vulnerability, unlike our baseline analysis, (23)], with younger age associated with higher levels of PGD symptoms. These associations between relationship with and age of the deceased are consistent with pre-pandemic studies (9, 10, 30, 53) and some studies of pandemic bereavement (8, 12–14, 29). Although our sample is not directly comparable to Aoun et al. (9) study, which provided empirical data for the proportions of low, moderate and high-risk groups reported above, comparisons of these participant characteristics (relationship with/age of deceased) would suggest that these two factors, albeit important predictors of grief severity, cannot explain the higher grief levels observed in our pandemic study. Mean age of deceased was very similar (72 vs. 75), while the Australian study included higher proportions of people who had lost spouses and children, and a similar proportion of people whose siblings had died.

### 4.3. Cause, expectedness, and place of death

While worse outcomes have been identified for people bereaved by COVID-19 compared with pre- pandemic general bereaved populations (12–14, 17), or “natural” causes of death before and during the pandemic (18–20), our analysis found no effect on PGD symptoms for cause of death (COVID-19 vs. non COVID-19) when other factors were controlled for (15), but a small and significant effect on vulnerability scores [unlike our baseline analysis, (23)]. However, it should also be noted that several of these factors/co-variates were associated with both COVID-19 deaths (22) and worse grief outcomes (discussed below, e.g., reduced support from healthcare professionals, loneliness and isolation). As in other studies, unexpected deaths were found to have a significant negative effect on PGD symptoms and grief vulnerability (19, 20, 50). The fact that a much larger group of participants reported that the death was not expected (78%) than those bereaved by COVID-19 (45%), would suggest that that this aspect of a death, which likely increased in relation to both COVID and non-COVID deaths during the pandemic [e.g., due to cancer treatment delays, disruption to services, (54, 55)] may be an explanatory factor for elevated PGD levels during the pandemic.

As in our baseline analysis (23), place of death was also found to be strongly associated with levels of grief. Despite care-home deaths being associated with worse experiences of end-of-life care and visiting restrictions (22), with the troubling consequences of prolonged periods of separation also described qualitatively (24), grief outcomes (IoV and PGD symptoms) were better when a death occurred in a care home compared with other settings (controlling for aspects of end-of-life experience, e.g., support from healthcare professionals after the death, being able to visit). This relationship may be due to anticipatory grief work, e.g., in the context of dementia diagnoses, and reflect the more “expected” nature of some of these deaths. A US study similarly found that deaths from dementia during the pandemic were negatively associated with probable PGD compared with deaths from other causes (18). The finding that those reporting “Other” places of death had worst PGD symptoms (although a very small group-size), followed by hospice and hospital deaths might reflect the consequences of especially traumatic, sudden deaths (e.g., accidents) (7, 19, 20), the more difficult end-of-life experiences identified in hospital settings, beyond those factors which we controlled for (22, 24), or distress and anger among relatives whose loved ones died of terminal illness, without the expected levels of treatment or care (24). The slightly better levels of social support perceived among those who experienced care home deaths, compared with hospital and hospice deaths, might also help to explain this relationship (35–37).

### 4.4. Circumstances of the death

There are a number of factors relating to the circumstances of the death that might be expected to have impacted upon grief levels. In previous quantitative and qualitative publications we identified experiences of sub-optimal end-of-life care, as healthcare systems and settings navigated the incredible strain and restrictions placed upon them (22, 24). As in our baseline analysis (23), we again found that feeling supported by healthcare professionals immediately following the death had a significant and lasting positive effect on PGD symptoms and grief vulnerability. Interestingly, we also found that this relationship with PGD symptoms was mediated by perceived social support at later time-points. This mediating effect might be explained by improved access to bereavement services as a result of supportive post-death care (and associated benefits relating to expressing feelings and receiving help with grieving, as captured in the ISS measure). It may also reflect the possibility that people with more negatives experiences (including problematic end-of-life care and related unanswered questions) may be more likely to feel poorly understood or unable to talk openly with others about how they are feeling, as also described in our qualitative findings (21, 24). Alongside other pandemic (17, 56, 57) and non-pandemic studies (44, 46) these findings demonstrate the importance of compassionate and effective communication around the time of death, and the likely significance of supportive post-death conversations and signposting for accessing further support and coming to terms with the circumstances of the death.

We found only small effects on PGD symptoms and grief vulnerability for factors relating to restricted contact at the end of life (e.g., visiting and saying goodbye), although being able to visit the patient at the end of life increased in importance once other factors were controlled in the PGD model, suggesting the “real” impact of this experience may be somewhat masked by other factors. These relatively small effects are nonetheless surprising given the devastating impacts of these experiences that were described in our qualitative data, including lasting feelings of guilt, anger, regret (24), and the effects of these circumstances identified in other studies (14, 15, 17, 20). “Dealing with my feelings around how my loved one died” was also the top-ranking need for support that we identified at baseline, with 60% of people experiencing high-level needs for help in this domain (21). These findings, the greater observed impact of feeling supported after the death, and examples in our interview data of some participants coming to terms with difficult end of life experiences (58), might therefore suggest that it is not just the circumstances of the death that are of importance, but also one’s ability to make sense of what happened (15, 58–60). This again points to the importance of good communication and support from healthcare providers for mitigating the effects of stressful and traumatic experiences (60).

### 4.5. Disruption to grieving, coping, and support processes

We found that loneliness and social isolation in early bereavement was strongly predictive of worse levels of PGD symptoms and grief vulnerability, as in our baseline analysis (23). Lower levels of social support, measured at later time points with the ISS, were also strongly predictive of poorer PGD symptoms. These findings are consistent with pandemic and pre-pandemic evidence on the negative impacts of social isolation, loneliness and lack of social support on bereavement outcomes (8, 29, 35–37). Our qualitative findings provide detailed accounts of how lockdown restrictions and shielding not only limited the emotional support and comfort available to people, but also prevented the collective rituals and acts of remembrance needed to begin processing their grief (21, 24). While the negative impacts of poor social support on grief is not unique to the pandemic, this is a factor clearly exacerbated by the pandemic-context, as people suffered not only from physical separation but also emotional “distance” and perceived lack of understanding and sensitivity to the realities of pandemic bereavement within social networks (15, 17, 21, 23, 24). Increased isolation and problematic social support during COVID-19 therefore seems likely to help explain elevated pandemic grief levels, while also reaffirming the important protective role of social support around the time of death and throughout bereavement generally [e.g., (10, 35–37)]. Positive support from friends and family described in our qualitative data included help with practical tasks, expressing feelings and sharing grief, remembering the person who died and feeling cared for and less isolated (61); benefits which are highly consistent with items in the social support measure that we used (43). Participants also commonly received emotional support from fellow bereaved people through online peer support communities and more formally from counselors, with both sources of support similarly valued for helping people to feel listened to and understood (61), further demonstrating how informal and formal emotional support can benefit people who are bereaved (62). Although restricted funerals had a large effect on PGD symptoms (and medium effect on grief vulnerability), this experience was not predictive once other factors were controlled for. This may be explained by the lessening effects of this experience over time and/or the greater relevance of other factors such as social support, along with the fact that almost all (93%) of respondents experienced these restrictions. Although most participants described the upsetting and distressing effects of restricted funerals in the qualitative data, a small minority described positive experiences and many described plans for future commemorative activities (24), which may have helped to mitigate the early effects of funeral restrictions. This uncertainty regarding the impact of funerals on bereavement outcomes is also reflected in the wider literature, which has been inconclusive (63) or found no effect (27), but also pointed to the important role of funeral providers and celebrants in providing alternative meaningful services in the contexts of restrictions.

### 4.6. Demographic influences and participant characteristics

Several demographic or participant factors were found to have an effect on support and grief outcomes. Existence of medical conditions was associated with higher levels of PGD symptoms (but not grief vulnerability), and a small negative (but non-significant) effect on perceived social support. This points toward the detrimental impact of poorer health status on a person’s ability to cope and adjust, in particular at a time where clinically vulnerable populations were required to “shield,” and opportunities for usual social and recreational activities and access to services were heavily restricted (24, 64). This is consistent with evidence from prior studies that existing mental-health conditions are associated with more complex grief (30, 65) or poorer mental health outcomes (66). There is of course also the possibility that the bereavement itself may have led to new or worsened medical conditions among some participants, indicated in the increased numbers of people reporting new conditions across time. This would be consistent with studies reporting increased rates of morbidity and mortality among surviving spouses compared with general populations [e.g., the “widowhood” effect, (67, 68)] and worse mental health among people bereaved during the pandemic compared with those not bereaved (66). Future analysis will investigate changes in health status and other associated factors over time (e.g., primary care and medication use) to explore these relationships further, along with other health economic outcomes, such as unemployment and time-off work.

As in previous research, and our baseline analysis (23), lower levels of education were associated with worse grief outcomes (PGD symptoms and grief vulnerability) (6, 30, 32–34). Although this factor became less important in the mixed-models, it underlines the importance of addressing structural disadvantage and inequity in healthcare and bereavement support (28, 69), particularly given the association that we previously identified between lower education-level and lower perceived healthcare professional support at the end of life (22). This association might reflect a poorer quality of healthcare in socially disadvantaged areas generally, e.g., due to higher demand and/or fewer resources [the “inverse care” law, (70, 71)], or the “cultural capital” and greater ability to engage and effectively communicate with healthcare providers among people from middle class backgrounds and with higher levels of formal education (72–74). This effect might also relate to the unequal impacts of the pandemic on poorer communities across the UK, potentially affecting community-level mental health and resilience, and in turn a more limited capacity for healthy grieving and adaptation among people living in the worst affected localities. The overall negative mental health impact of living through the pandemic at general population-level has been documented (66, 75, 76), including worse outcomes associated with lower socio-economic status (8, 76), economic stressors (77), and bereavement (66, 77).

Despite the disproportionate impacts of the pandemic on minoritized ethnic communities, in terms of death-rates, and disruption to grieving practices and community networks (28, 78), this group of participants actually had better levels of PGD symptoms (but not grief vulnerability), although the difference was not statistically significant in the mixed-model. Further, the small size of this group (particularly at later time points) and the lessened effect of ethnicity once other factors were controlled, means that this finding should be treated cautiously. Of note though, is the observed potential mediating role of social support, and possibility that the better social support reported by our minority ethnic participants may have mitigated the effects of some of the general and culturally-specific challenges of pandemic bereavement faced by minoritized communities [e.g., see (21, 28, 75)], although again these differences should be treated with caution.

### 4.7. The bigger picture

This analysis has identified several pandemic-related factors which, in influencing grief outcomes, might at least partially explain the apparently higher levels of indicated PGD that we observed, compared with similar non-pandemic studies (9). When considered alongside our qualitative findings, however, what is also apparent is the intensity of feelings surrounding these and other factors; experiences which clearly had compounding and far-reaching effects on the lives of our participants, but which were not fully captured in our quantitative measures and analyses. Pandemic-related factors not fully measured or included in this analysis, but which we know to be highly consequential for grieving include: death-trauma (e.g., perceived suffering, poor treatment, shock), inability to collectively mourn or remember loved ones, the isolating and disenfranchising effects of being bereaved during a prolonged period of mass-bereavement (including lasting anger at political and societal responses to the pandemic and continuing fear of the virus), limited opportunities to engage in recreational and other coping activities, stressful death-administration and financial/work-based challenges, and reduced access to critical support-services (21, 24).

Within our qualitative findings, as in other studies (15), the significance of meaning-making in mediating the effects of many of these circumstantial factors is also evident (21, 24, 58). Examples of pandemic-related difficulties finding meaning included anger and unanswered questions surrounding and preventing acceptance of the death, descriptions of grief feeling “unreal” without recourse to collective ritual, and lack of appropriate support and help with processing feelings (21, 24). Pandemic-related disruption to meaning-making processes might therefore also explain the higher levels of pathological grief that we observed, as well as the small or insignificant effects of factors where only the occurrence of the “event” rather than responses to it was captured (e.g., restricted funerals, being unable to say goodbye). This underlines the importance not only of considering how any infection-control restrictions that may be needed are managed and implemented (with meaningful alternatives available where possible), but equally that there is appropriate and effective communication with bereaved people and support surrounding any restrictions, coupled with opportunities to formally revisit and reflect upon what happened.

### 4.8. Strengths and weaknesses

This longitudinal study benefits from a large initial sample-size, with quantitative and qualitative data collected across four time points up to approximately 2 years post-bereavement. Although participant numbers decreased over this time-period, we retained sufficient numbers to enable robust analysis, albeit with reducing proportions of younger and older participants, and people from minoritized ethnic backgrounds or with lowest qualifications levels. The sample was reasonably well represented across geographical areas, education and deprivation, but was self-selecting and biased toward female and white respondents, despite targeting men and people from minoritized ethnic communities in our recruitment approaches. Due to missing data on religious/spiritual beliefs and sexual orientation we were also unable to consider the effects of these other aspects of participant identity and subjectivity that may have influenced their coping and grief experiences (27, 32, 79, 80). By recruiting mostly online, we were less likely to reach the very old or other digitally marginalized groups. Convenience sampling might have resulted in more people with negative experiences participating, as well as those accessing support. Despite these limitations, group sizes were sufficient to enable comparisons (although not to the level of specific ethnic groups) and, while not providing population-level prevalence data, the sample does enable comparisons to be made with data from similar pre-pandemic studies [e.g., (9)], and the identification of potential risk factors which can inform future practice and policy. However, as argued elsewhere, there is a risk of pathologizing “normal” grief when attempting to determine PGD using fixed time-frames (81), especially in a pandemic context when “delayed grief” has been hypothesized (82, 83), further underlining the importance of collecting longitudinal and long-term follow-up data.

### 4.9. Implications for further research

Through subsequent qualitative interviews, we have explored in depth the experiences of people with characteristics less well represented in the survey, including men, people identifying with a sexual or ethnic minority background, with publication forthcoming. However, further research is required exploring the needs of bereaved people from minoritized ethnic and socially disadvantaged backgrounds, same-sex couples, men, children and young people, and people with pre-existing mental health conditions, as we navigate the COVID-recovery phase and beyond. Research exploring the impact of religious and spiritual beliefs would also help to address current gaps in the evidence (27). Given the importance of our qualitative data for establishing the “bigger picture” and the meaning of pandemic circumstances for bereaved individuals, the use of qualitative or mixed-methods approaches when investigating novel and unpredictable future mass-bereavement events is essential. The development or further refinement of tools for measuring identified event-specific risk factors, and the meaning and significance of such factors for individuals, e.g., the Inventory of Pandemic Grief Risk Factors (17), would also be helpful.

## 5. Conclusions and implications for policy and practice

We found relatively high-levels of indicated PGD at c. 8, 13, and 25 months post-bereavement when compared with similar non-pandemic studies of bereaved populations [e.g., (9)]. Several pandemic-related factors were identified which, in influencing grief outcomes, seem likely to at least partially explain this phenomenon. The strongest of these predictors were social isolation and problems accessing social support during bereavement, which while not unique to the pandemic was almost certainly exacerbated by it (21). Other likely explanatory factors included higher rates of unexpected deaths, and the disproportionately higher numbers of deaths occurring within socially deprived/less formally “educated” communities during the pandemic (given the poorer grief outcomes of these groups). In their relationships with grief-levels, and the unique pandemic-context, poorer care-experiences at the end of life (including visiting restrictions) and the existence of other medical conditions, might also help to explain higher grief levels among people bereaved during the pandemic.

However, effect sizes for many of these factors were in absolute terms “small,” and our qualitative insights paint a much fuller and more intricate picture than we could capture in our quantitative measures and analyses. Taken together, our mixed-methods findings suggest that is likely the combined and compounding effects of the many different challenging experiences of people bereaved during the pandemic that contributed to higher-levels of complex and prolonged grief.

Based on these findings we make the following recommendations to inform bereavement support and policy at the present time and in future pandemics, many of which resonate with the recent report by the UK Commission on Bereavement (78).

Implications for the current COVID-recovery phase and beyond:

1. In view of the higher proportions of people experiencing or at risk of PGD following the pandemic, bereavement support services require increased investment to ensure adequate levels of specialist provision which can effectively cater for those with more complex needs, as well as robust methods of identifying and reaching people most in need of more intensive support. Bereaved people more likely to require such support include those grieving children, partners and siblings and following unexpected deaths, as well as people who are isolated and have limited social support, health conditions and low levels of formal education.
2. Opportunities for informal emotional and social support should be strengthened through provision of peer-support groups, as well as compassionate community initiatives and educational programs which seek to improve grief literacy and the support available to people within existing social and community networks (84). Communities worst affected by COVID-19 and structural inequalities should be prioritized for such initiatives.
3. Policies and training should be implemented to ensure compassionate and supportive communication and behaviors from healthcare professionals at the end of life, especially in acute and care-home settings. “Follow-up” contact should be consistently delivered by care providers following the death and enable meaningful discussion and reflection on difficult and troubling experiences, with signposting to locally and nationally available bereavement support services.

To ensure preparedness for future pandemics and other mass-bereavement events, best practice-guidance and related policies should be developed for:

4. Health-care settings, with specific regard to managing and balancing infection-risk with the need to facilitate patient-family contact, including use of Personal Protective Equipment and remote communication-methods, and ensuring effective and compassionate communication with family members during times of crises.

5. Funeral-providers and crematoria, including identifying different options for meaningful and alternative funeral and mourning practices when restrictions are needed. The role of funeral directors in providing compassionate and supportive responses should be recognized, including their roles in sign-posting to further support-services (27, 62).

6. Managing social contact, recognizing the need to restrict social interaction in times of high-infection rates, while making allowances for those living alone and with particular vulnerabilities, including the recently bereaved. Greater understanding of permissible levels of “safe” contact relative to infection levels, and the best means of enabling this (e.g., outdoor socialization) would also be helpful.

7. Rapid mobilization of locally and nationally coordinated bereavement support provision, including existing providers and other community organizations. Any such responses should involve proactive sign-posting to and advertising of such support, mechanisms for identifying those requiring more intensive specialist support, and crisis-specific training and practice- sharing to ensure that the support offered is crisis- as well as culturally-competent (21, 85).

## Data availability statement

The datasets presented in this study can be found in online repositories. The names of the repository/repositories and accession number(s) can be found at: UK Data Service via https://reshare.ukdataservice.ac.uk/855751.

## Ethics statement

The studies involving humans were approved by the Cardiff University School of Medicine Research Ethics Committee. The studies were conducted in accordance with the local legislation and institutional requirements. The participants provided their written informed consent to participate in this study.

## Author contributions

EH and LS designed the study, led the application for funding, and were co-principal investigators. EH drafted the manuscript. RM conducted the statistical analyses, with data management assistance from SG. RM wrote the statistical analysis and results sections of the manuscript. SG, ML, AB, DF, LM, KS, AP, and SS were members of the research team or the study advisory group and contributed to the design of the study and survey. All authors contributed to drafting the manuscript and read and approved the final manuscript.

## Funding

This study was funded by the UKRI/ESRC (Grant No. ES/V012053/1), with the final fourth survey round funded by a Marie Curie Small Grant (MCSGS-21-701). This project was also supported by the Marie Curie core grant funding to the Marie Curie Research Centre, Cardiff University (grant no. MCCC-FCO-11-C). EH, AB, SS, and LM were supported by the Marie Curie core grant funding (grant no. MCCC-FCO-11-C). The funder was not involved in the study design, implementation, analysis or interpretation of results and has not contributed to this manuscript.

## Conflict of interest

AP declared a potential financial interest relating to lobbying by the Childhood Bereavement Network and National Bereavement Alliance for additional financial support for the bereavement sector.

The remaining authors declare that the research was conducted in the absence of any commercial or financial relationships that could be construed as a potential conflict of interest.

## Publisher’s note

All claims expressed in this article are solely those of the authors and do not necessarily represent those of their affiliated organizations, or those of the publisher, the editors and the reviewers. Any product that may be evaluated in this article, or claim that may be made by its manufacturer, is not guaranteed or endorsed by the publisher.

## Footnotes

^1^https://www.onlinesurveys.ac.uk/

^2^www.covidbereavement.com

^3^www.r-studio.com
