# Supplementary material for: Prolonged grief during and beyond the pandemic: factors associated with levels of grief in a four time-point longitudinal survey of people bereaved in the first year of the COVID-19 pandemic
Source: Front Public Health. 2023 Sep 19;11:1215881. doi: 10.3389/fpubh.2023.1215881 (PMC10546414; doi:10.3389/fpubh.2023.1215881)
Supplement: Supplementary file 3 [file Table_1.docx]

Table S1. Full list of additional R packages used in the statistical analysis
